# Co-display of diverse spike proteins on nanoparticles broadens sarbecovirus neutralizing antibody responses

**DOI:** 10.1016/j.isci.2022.105649

**Published:** 2022-11-22

**Authors:** Mitch Brinkkemper, Tim S. Veth, Philip J.M. Brouwer, Hannah Turner, Meliawati Poniman, Judith A. Burger, Joey H. Bouhuijs, Wouter Olijhoek, Ilja Bontjer, Jonne L. Snitselaar, Tom G. Caniels, Cynthia A. van der Linden, Rashmi Ravichandran, Julien Villaudy, Yme U. van der Velden, Kwinten Sliepen, Marit J. van Gils, Andrew B. Ward, Neil P. King, Albert J.R. Heck, Rogier W. Sanders

**Affiliations:** 1 Amsterdam UMC, location University of Amsterdam, Department of Medical Microbiology and Infection Prevention, Laboratory of Experimental Virology, Meibergdreef 9, 1105 AZ Amsterdam, the Netherlands; 2 Amsterdam Institute for Infection and Immunity, Infectious Diseases, Amsterdam, the Netherlands; 3 Biomolecular Mass Spectrometry and Proteomics, Bijvoet Center for Biomolecular Research and Utrecht Institute for Pharmaceutical Sciences, University of Utrecht, Padualaan 8, 3584 CH Utrecht, the Netherlands; 4 Netherlands Proteomics Center, Padualaan 8, 3584 CH Utrecht, the Netherlands; 5 Department of Integrative Structural and Computational Biology, The Scripps Research Institute, La Jolla, CA 92037, USA; 6 Department of Biochemistry, University of Washington, Seattle, WA 98195, USA; 7 Institute for Protein Design, University of Washington, Seattle, WA 98195, USA; 8 J&S Preclinical Solutions, 5345 RR, OSS, the Netherlands; 9 AIMM Therapeutics BV, 1105 BA Amsterdam, the Netherlands; 10 Department of Microbiology and Immunology, Weill Medical College of Cornell University, New York, NY 10065, USA

**Keywords:** Immunology, Immune response, Microbiology, Virology

## Abstract

The emergence of severe acute respiratory syndrome coronavirus 2 (SARS-CoV-2) variants poses continuous challenges in combating the virus. Here, we describe vaccination strategies to broaden SARS-CoV-2 and sarbecovirus immunity by combining spike proteins based on different viruses or viral strains displayed on two-component protein nanoparticles. First, we combined spike proteins based on ancestral and Beta SARS-CoV-2 strains to broaden SARS-CoV-2 immune responses. Inclusion of Beta spike improved neutralizing antibody responses against SARS-CoV-2 Beta, Gamma, and Omicron BA.1 and BA.4/5. A third vaccination with ancestral SARS-CoV-2 spike also improved cross-neutralizing antibody responses against SARS-CoV-2 variants, in particular against the Omicron sublineages. Second, we combined SARS-CoV and SARS-CoV-2 spike proteins to broaden sarbecovirus immune responses. Adding SARS-CoV spike to a SARS-CoV-2 spike vaccine improved neutralizing responses against SARS-CoV and SARS-like bat sarbecoviruses SHC014 and WIV1. These results should inform the development of broadly active SARS-CoV-2 and pan-sarbecovirus vaccines and highlight the versatility of two-component nanoparticles for displaying diverse antigens.

## Highlights

• Two-component nanoparticles are very suitable for co-displaying diverse proteins
• A third vaccination with SARS-CoV-2 spike improves responses against Omicron variants
• Co-display of SARS-CoV-2 ancestral and Beta spikes improves neutralizing antibody responses against variants of concern
• Co-display of SARS-CoV and SARS-CoV-2 spikes improves sarbecovirus neutralizing responses

## Graphical abstract

**Figure:**
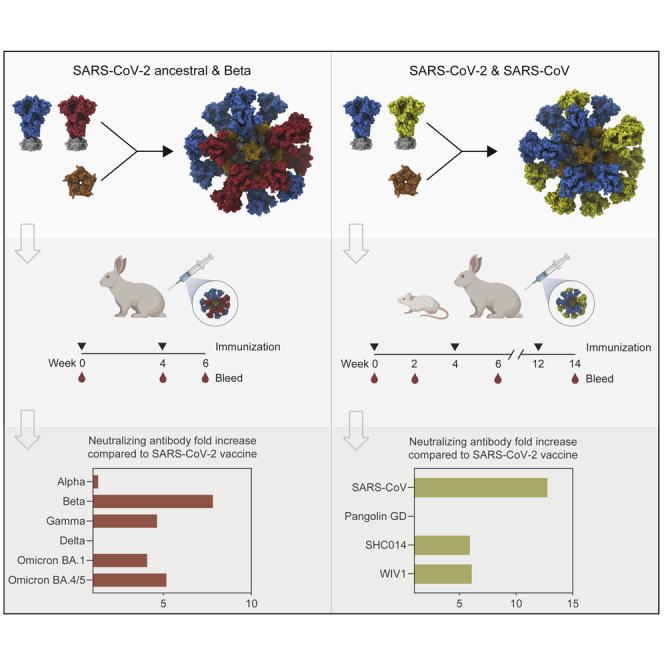

## Introduction

In late 2019, severe acute respiratory syndrome coronavirus 2 (SARS-CoV-2) first appeared and quickly spread across the globe, causing the coronavirus disease 2019 (COVID-19) pandemic. COVID-19 generally manifests itself as a mild respiratory disease but can cause acute respiratory distress syndrome, which leads to death in a significant number of cases.^1^ As of October 1, 2022, at least 600 million people have been infected and 6.5 million have died of COVID-19 (https://covid19.who.int/). After the discovery of SARS-CoV-2, the production of a safe and effective vaccine quickly became top priority. Currently, multiple vaccines have been approved by the US Food and Drug administration, European Medicines Agency, and other regulatory agencies and have been rolled out.^2^^,^^3^^,^^4^^,^^5^

Neutralizing antibodies (NAbs) are the most prominent correlate of the protection of SARS-CoV-2 vaccines,^6^ and many SARS-CoV-2 NAbs have been isolated from COVID-19 patients, which all bind the spike (S) protein of the virus.^7^^,^^8^^,^^9^^,^^10^ S interacts with the host angiotensin-converting enzyme 2 (ACE2) receptor to enable merging of the viral envelope with the cell membrane.^11^ The main targets for NAbs are the receptor-binding domain (RBD) and the N-terminal domain (NTD) of S.^7^ Vaccines currently in use often deploy a proline stabilized prefusion S protein based on the ancestral Wuhan-Hu-1 strain.^12^ The emergence of SARS-CoV-2 variants that contain one or multiple mutations in the RBD and NTD cause these vaccines to be less effective in preventing infection. Indeed, SARS-CoV-2 variants, especially Beta and Omicron, are less sensitive to neutralization by sera from convalescent patients and vaccinated individuals.^13^^,^^14^^,^^15^^,^^16^^,^^17^^,^^18^ Furthermore, SARS-CoV-2 variants, in particular Delta and Omicron, show progressively increased transmissibility.^19^^,^^20^ Booster vaccines specifically targeting SARS-CoV-2 variants are currently being tested in phase 1 and 2 clinical trials,^21^^,^^22^ but vaccines that incorporate sequence diversity could improve and broaden protection against new SARS-CoV-2 strains.

Multimeric presentation of antigens is a well-established strategy for inducing strong humoral immune responses. Immunological processes such as retention on follicular dendritic cells, lymph node trafficking, and strong B cell activation are aided by nanoparticles (NPs) presenting a repetitive array of antigen.^23^^,^^24^ Previously, we described a SARS-CoV-2 S-I53-50 two-component protein NP vaccine that induced potent NAb responses in multiple animal models and protection against SARS-CoV-2 challenge in cynomolgus macaques.^25^ Two-component protein NPs such as I53-50 can be assembled *in vitro*, allowing for separate production of individual subunits followed by rigorous quality control before particle assembly. We and others have used this platform to induce strong humoral immune responses against respiratory syncytial virus (RSV),^26^ influenza,^27^ hepatitis C virus^28^, Lassa virus,^29^ and HIV-1.^30^ I53-50 NPs are in clinical phase testing for RSV, and an I53-50-based COVID-19 vaccine was recently approved for use in South Korea.^31^

In this study, we exploited the I53-50 NP platform to present S proteins from both the ancestral SARS-CoV-2 and Beta strains and evaluated the immunogenicity in rabbits. In a next iteration, we produced I53-50 NPs that present both SARS-CoV S and SARS-CoV-2 S to induce broad sarbecovirus responses in mice and rabbits. We compared these data to our previous studies in which we immunized mice and rabbits with soluble SARS-CoV-2 S and SARS-CoV-2 S-I53-50 NPs.^25^^,^^32^

## Results

### Ancestral and Beta SARS-CoV-2 spikes can be displayed on I53-50 NPs

**Figure 1:**
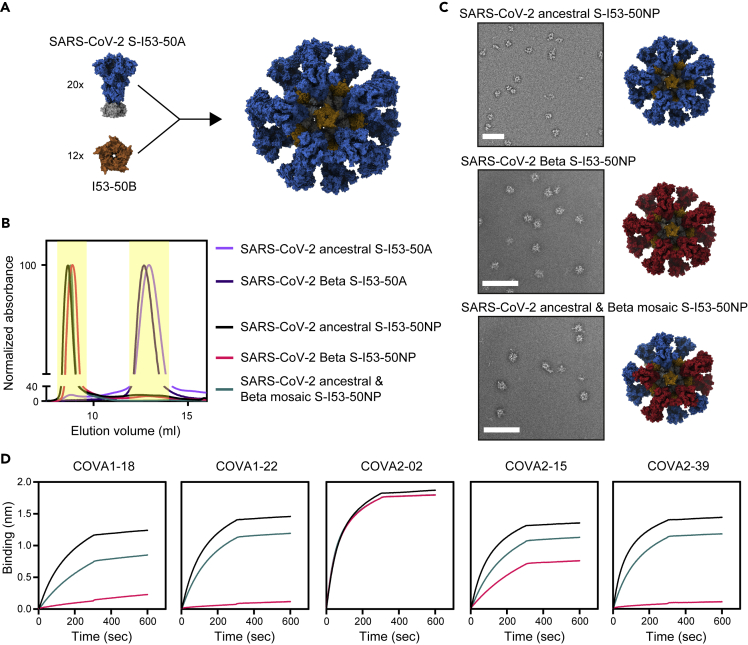
Biophysical and antigenic characterization and mass spectrometry analysis of SARS-CoV-2 S-I53-50 NPs (A) Schematic representation of 20 SARS-CoV-2 S-I53-50A (SARS-CoV-2 S in blue and I53-50A in gray) and 12 I53-50B assembling into SARS-CoV-2 S-I53-50 NP. (B) Size exclusion chromatograms of S-I53-50A.1NT1 proteins and S-I53-50 NPs run over a Superose 6 Increase 10/300 GL column. The graph shows an overlay of the different runs. The yellow columns specify the SEC fractions that were collected and pooled. (C) Negative stain EM analysis of assembled S-I53-50 NPs. The white bar represents 200 nm. (D) BLI sensorgrams showing the binding of multiple SARS-CoV-2 NAbs to S-I53-50 NPs.

The computationally designed two-component I53-50 NP platform is well suited for the display of diverse S proteins. I53-50 consists of 20 trimeric components (I53-50A or variations thereof; the “A component”) and 12 pentameric components (I53-50B.4PT1; the “B component”) that together assemble into monodisperse icosahedral particles (Figure 1A).^33^ We previously presented the ancestral SARS-CoV-2 S protein on I53-50 NPs.^25^ Here, our aim was to display Beta S as well as the combination of ancestral and Beta S. Accordingly, we genetically fused the N-terminus of the A component to the C-terminus of the proline stabilized prefusion Beta S protein using a glycine-serine linker. The S-A component fusion proteins were harvested from the supernatant of transfected human embryonic kidney (HEK) 293F cells and purified using Ni-NTA affinity columns. After affinity purification, constructs were refined using size exclusion chromatography (SEC) and collection of the appropriate fractions (Figure 1B). Particles were assembled by mixing the purified S-A component fusion proteins with equimolar amounts of the B component and incubating overnight at 4°C. These mixtures were then subjected to an additional SEC step to remove unassembled components and stored at −80°C (Figure 1B). Mosaic NPs were produced by mixing the appropriate S-A component fusion proteins at equimolar amounts before adding the B component. Negative stain electron microscopy (nsEM) confirmed the presence of assembled NPs (Figure 1C).

A bio-layer interferometry (BLI)-based assay using a panel of monoclonal Abs (mAbs) previously isolated from COVID-19 patients was used to confirm the presence of intact S epitopes and, in mosaic NPs, the presence of multiple different S proteins (Figure 1D). RBD-specific COVA1-18, COVA2-02, COVA2-15, and COVA2-39 and NTD-specific COVA1-22 showed strong binding to ancestral SARS-CoV-2 S-NP. Ancestral SARS-CoV-2-specific COVA1-18, COVA1-22, and COVA2-39 showed no binding to SARS-CoV-2 Beta S-NP, while showing intermediate binding to the mosaic S-NP. COVA2-15, which has decreased binding to Beta S compared with ancestral SARS-CoV-2 S, showed intermediate binding to SARS-CoV-2 Beta S-NP and the mosaic S-NP. COVA2-02 is a broadly binding Ab that showed binding to all particles equally, as was expected. Together, the SEC, nsEM, and BLI data confirmed the production of NPs that present multiple copies of S and show that our mosaic preparations contain a mixture of S proteins.

While these experiments did not provide formal proof that ancestral and Beta S were present on the same NPs, we do provide such evidence below for more distinct S proteins from SARS-CoV and SARS-CoV-2.

### Inclusion of Beta S improves NAb responses against some variants in rabbits

**Figure 2:**
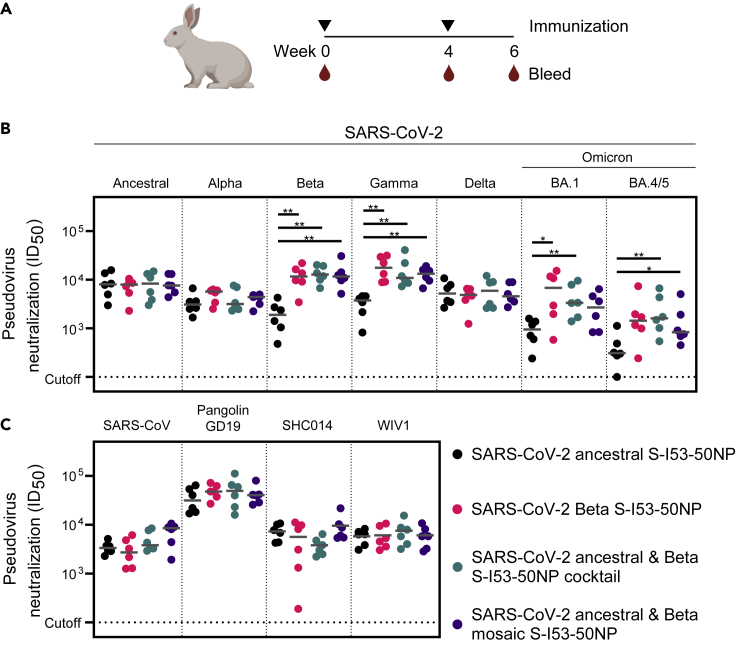
Immunogenicity of SARS-CoV-2 S and SARS-CoV-2 Beta S in rabbits (A) Study schedule in rabbits. Black triangles indicate immunizations and drops indicate bleeds. (B) SARS-CoV-2 ancestral, Alpha, Beta, Gamma, Delta, and Omicron BA.1 and BA.4/5 pseudovirus neutralization at week 6. (C) SARS-CoV, Pangolin GD, SHC014, and WIV1 pseudovirus neutralization at week 6. (B and C) The median titers are indicated by a bar. Titers between groups were compared using the Mann-Whitney *U* test (∗, p < 0.05; ∗∗, p < 0.01).

Six New Zealand White rabbits per group were immunized with 15 μg ancestral SARS-CoV-2 S-NP, SARS-CoV-2 Beta S-NP, a cocktail of ancestral SARS-CoV-2 S-NP and SARS-CoV-2 Beta S-NP, or a mosaic NP displaying both S proteins. All NPs were adjuvanted with squalene emulsion.^34^ Animals were immunized at weeks 0 and 4, and animals were bled at weeks 0, 4, and 6 (Figure 2A). Neutralization of ancestral SARS-CoV-2 (B.1; D614G) and the variants of concern (VOC), Alpha, Beta, Gamma, Delta, and Omicron BA.1 and BA.4/5 at all bleeds was measured in a pseudovirus neutralization assay (Figure 2B and Table S1). We also measured neutralization of SARS-CoV and animal coronaviruses Pangolin GD, SHC014, and WIV1 (Figure 2C and Table S1). Pangolin GD is a sarbecovirus isolated from the Malayan pangolin^35^ and is closely related to SARS-CoV-2 and SARS-CoV with 89.8 and 76.8% amino acid (AA) sequence identity in the S protein, respectively. SHC014 and WIV1 are sarbecoviruses that were isolated from the Chinese horseshoe bat^36^ and have 77.0% and 71.9% AA sequence identity in the S protein with SARS-CoV-2 and 89.9% and 74% with SARS-CoV S, respectively.

Ancestral S-NP induced the strongest NAb responses, which are represented as inhibitory dilutions at which 50% neutralization is attained (ID_50_ values), against the ancestral strain and only slightly weaker responses against the Alpha, Gamma, and Delta strains two weeks after the second immunization (median ID_50_ values of 8,073, 3,089, 3,758, and 5,215, respectively) (Figure 2B). The neutralization titers against Beta and Omicron BA.1 and BA.4/5 were substantially lower (median ID_50_ values of 1,887, 946, and 309, respectively). The Beta, cocktail, and mosaic vaccines induced similar neutralization titers against ancestral, Alpha, and Delta strains compared to the ancestral S-NP immunizaed animals. However, titers against Beta, Gamma, and Omicron BA.1 and BA.4/5 were 6.2-, 4.7-, 7.3-, and 4.6-fold higher in the Beta S-NP immunized animals compared with the ancestral S-NP group (median ID_50_ values of 11,646 versus 1,887, p = 0.0043; 17,724 versus 3,758, p = 0.0022; 6,847 versus 946, p = 0.0411; 1,434 versus 309, p = 0.0649, respectively) (Figure 2B). This difference was already apparent after one immunization (Table S1). All groups developed significant levels of neutralization against sarbecoviruses SARS-CoV, Pangolin GD, SHC014, and WIV1. The mosaic NPs induced higher responses against SARS-CoV than any of the other groups, although the difference was not statistically significant (median ID_50_ values of 8,517 versus 3,817, p = 0.1797, in the mosaic and cocktail groups, respectively). The responses against Pangolin GD, SHC014, and WIV1 were similar in all groups (ID_50_ values around 42,000, 6,500, and 6,000, respectively) (Figure 2C).

### SARS-CoV-2 and SARS-CoV spikes can be co-displayed on I53-50 NPs

**Figure 3:**
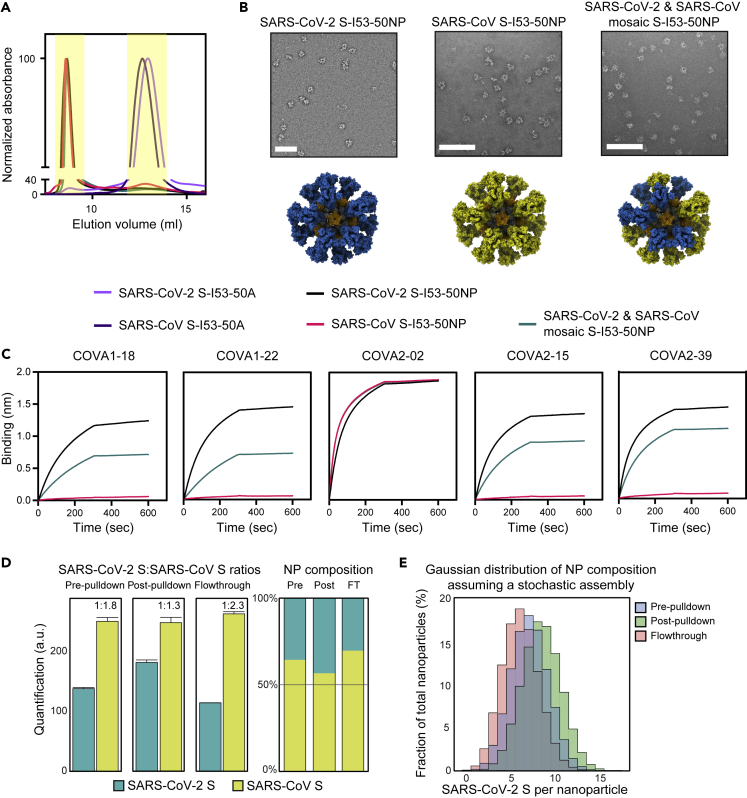
Biophysical and antigenic characterization and mass spectrometry analysis of SARS-CoV-2 and SARS-CoV S-I53-50 NPs (A) Size exclusion chromatograms of S-I53-50A.1NT1 proteins and S-I53-50 NPs run over a Superose 6 Increase 10/300 GL column. The graph shows an overlay of the different runs. The yellow columns specify the SEC fractions that were collected and pooled. (B) Negative stain EM analysis of assembled S-I53-50 NPs. The white bar represents 200 nm. (C) BLI sensorgrams showing the binding of multiple SARS-CoV-2 NAbs to S-I53-50 NPs. (D) Quantification of SARS-CoV S and SARS-CoV-2 S on mosaic NPs in the pre-pull-down, post-pull-down, and flow-through fractions (pull-down with SARS-CoV-2-specific COVA1-18), using SRM assays based on unique peptides. The abundance of the SARS-CoV S is normalized against the (constant) nanocomponent I53-50A.1NT1. (E) Gaussian distribution calculated based on the mean number of SARS-CoV S and SARS-CoV-2 S assuming a stochastic assembly.

I53-50 NPs displaying SARS-CoV S as well as the combination of SARS-CoV-2 and SARS-CoV S were produced similarly to the NPs described previously. The N-terminus of the I53-50 A-component was genetically fused to the C-terminus of the proline stabilized prefusion SARS-CoV S protein using a glycine-serine linker. Proteins were harvested from the supernatant of transfected HEK 293F cells and purified using Ni-NTA affinity columns. After affinity purification, constructs were further purified using SEC (Figure 3A), particles were assembled, and subjected to an additional SEC step as described previously (Figure 3A). Mosaic NPs were produced by mixing the appropriate S-A component fusion proteins at equimolar amounts before assembly. Negative stain EM confirmed the presence of assembled NPs (Figure 3B).

With the same mAb panel as described previously, a BLI-based assay was performed to confirm the presence of intact S epitopes and in mosaic NPs the presence of multiple different S proteins (Figure 3C). RBD-specific COVA1-18, COVA2-02, COVA2-15, and COVA2-39 and NTD-specific COVA1-22 showed strong binding to SARS-CoV-2 S-NP. SARS-CoV-2-specific COVA1-18, COVA1-22, COVA2-15, and COVA2-39 showed no binding to SARS-CoV S-NP, while showing intermediate binding to the mosaic S-NP. COVA2-02 is a broadly binding Ab that showed binding to all particles equally, as was expected. These data together confirmed the production of NPs that present multiple copies of S and show that our mosaic S-NP samples contain a mixture of S proteins.

The former experiments did not formally prove that the different S proteins were present on the same NPs in the mosaic preparations. In theory, there is a possibility that different S proteins fused to the A component might preferentially assemble into homotypic NPs, not mosaic NPs. To assess whether our mosaic S-NPs actually presented a mixture of the two S proteins, we set up a targeted mass spectrometry-based selected reaction monitoring (SRM) assay, enabling the quantitative measurement of SARS-CoV-2 S and SARS-CoV S protein in the mosaic NP sample. We applied this assay to the mosaic NPs before and after a SARS-CoV-2 specific pull-down with the COVA1-18 mAb. In short, COVA1-18 was coupled to beads bound with protein A, and a pull-down was performed on the mosaic NP sample. NPs were eluted from the beads and digested using trypsin and LysC. After digestion, heavy-labeled proteotypic AQUA peptides were added to the mix of endogenous NP-derived peptides. Using the SRM assay, the ratio between the heavy-labeled peptides and the endogenous peptides was accurately determined and used to quantify molar concentration of all proteins in the mosaic NPs before and after the pull-down, also evaluating the flow-through samples.

SRM experiments determined that the SARS-CoV-2:SARS-CoV S ratio in the mosaic NP sample before the pull-down was 1:1.8, implying that SARS-CoV S was incorporated into NPs with a somewhat greater efficiency compared with SARS-CoV-2 S. After pull-down with COVA1-18, the ratio dropped significantly to 1:1.3, indicative of selective enrichment for SARS-CoV-2 S. The reverse was true for the flow-through, where the SARS-CoV-2:SARS-CoV S ratio was 1:2.3, supporting the enrichment of SARS-CoV-2 S by the COVA1-18 pull-down (Figure 3D). The pull-down with COVA1-18 was clearly incomplete as NPs that contain SARS-CoV-2 S were present in the flow-through. It is possible that NPs with only one or a few SARS-CoV-2 S proteins were not captured efficiently by the protein A-COVA1-18 beads because of the lack of avidity. Nevertheless, the pull-down experiment showed that mosaic NPs displaying both SARS-CoV-2 and SARS-CoV S were formed.

A homogeneous mosaic population would have identical quantities of SARS-CoV-2 and SARS-CoV S protein in all the analyzed samples. The shift in SARS-CoV-2 and SARS-CoV S quantities in the pull-down experiment is therefore indicative of a heterogeneous distribution. Assuming that the NPs assemble stochastically, whereby the stoichiometry distribution is determined by the relative abundance and/or assembly efficiency of the two proteins, the population heterogeneity can be calculated.^37^ A Gaussian distribution was calculated based on the mean number of SARS-CoV-2 S and SARS-CoV S before and after pull-down and in the flow-through (Figure 3E). The distribution of the three samples clearly separate, but while the stoichiometry distributions differ in each sample, all three show a vast majority of NPs containing both SARS-CoV-2 S and SARS-CoV S and negligible amounts of almost uniquely SARS-CoV-2 S or SARS-CoV S containing NPs.

### Cocktail and mosaic NPs displaying SARS-CoV-2 and SARS-CoV S induce broad sarbecovirus Ab responses

**Figure 4:**
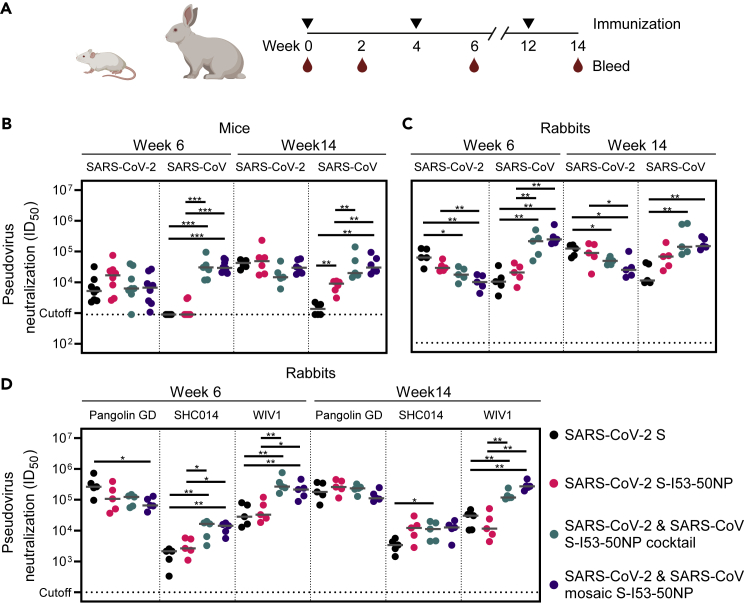
Immunogenicity of SARS-CoV S and SARS-CoV-2 S in mice and rabbits (A) Study schedule in mice and rabbits. Black triangles indicate immunizations and drops indicate bleeds. (B) SARS-CoV-2 and SARS-CoV pseudovirus neutralization in mice. For mice sera, a cutoff of 900 was used due to low sample availability. (C) SARS-CoV-2 and SARS-CoV pseudovirus neutralization in rabbits. (D) SARS-CoV, Pangolin GD, SHC014, and WIV1 pseudovirus neutralization in rabbits. (B–D) The median titers are indicated by a bar. Titers between groups were compared using the Mann-Whitney *U* test (∗, p < 0.05; ∗∗, p < 0.01; ∗∗∗, p < 0.001).

The immunogenicity of mosaic and cocktail NPs was evaluated in both mice and rabbits. Eight BALB/c mice were immunized with 10 μg of a cocktail of SARS-CoV S-NP and SARS-CoV-2 S-NP or a mosaic S-NP displaying both S proteins. All NPs were adjuvanted in polyinosinic-polycytidylic acid (Poly-IC). Five New Zealand White rabbits were immunized with 30 μg of the same cocktail and mosaic S-NP. NPs were adjuvanted with squalene emulsion. Animals were immunized at weeks 0, 4, and 12 and were bled at week 0 and 2 weeks after each immunization (Figure 4A). For comparison, we reanalyzed sera from previous studies in which mice and rabbits were immunized with soluble SARS-CoV-2 S and SARS-CoV-2 S-NP using the same immunization regimen.^25^^,^^32^

Ab binding was analyzed to assess the induction of cross-binding Abs. These experiments were performed with only the rabbit samples, because of low availability of the mouse sera. All immunogens induced high binding Ab titers against SARS-CoV-2 S after three immunizations in rabbits. SARS-CoV-2 S and S-NP induced the highest binding titers (median serum dilution endpoint titers of ∼250,000 for both groups), whereas titers in the cocktail and mosaic S-NP groups were slightly lower (medians of 165,915 and 90,159, respectively) (Figure S1A). SARS-CoV-2 S and S-NP induced significantly lower Ab binding against SARS-CoV S (median titers of 38,150 and 63,326, respectively) compared with the cocktail and mosaic S-NP groups (median titers of 184,724 and 130,062, respectively), consistent with the presence of SARS-CoV S in the cocktail and mosaic groups. Next, to assess whether immunizations with the cocktail and mosaic S-NPs induced cross-binding Abs, we conducted a depletion ELISA (Figure S1B). S-specific Ab in rabbit serum samples from week 14 were depleted with either SARS-CoV S or SARS-CoV-2 S and Ab binding against the other S protein was tested by ELISA. In groups immunized with soluble SARS-CoV-2 S and SARS-CoV-2 S-NP, SARS-CoV S binding could be almost completely depleted with SARS-CoV-2 S, as would be expected. In the same groups, Ab binding to SARS-CoV-2 S was depleted by 32% and 54% by SARS-CoV S, respectively. Abs induced by the cocktail and mosaic NPs were depleted for 50% or more by both SARS-CoV-2 S and SARS-CoV S when looking at binding to the other S. Overall, these data show that the cocktail and mosaic S-NPs induce SARS-CoV-2 S and SARS-CoV S cross-binding Abs.

SARS-CoV-2 S-NPs induced approximately 3-fold higher neutralization titers against SARS-CoV-2 (median ID_50_ of 16,792) compared with the other immunogens in mice at week 6, although the difference did not reach statistical significance. Titers in the SARS-CoV-2 S-NP group improved by 3-fold (median ID_50_ value of 49,031, p = 0.2188) after the third immunization, but this was again not statistically significant, while titers in the other groups were similar or slightly lower. The cocktail and mosaic S-NPs induced the most potent neutralization against SARS-CoV at week 6 (median ID_50_ values of ∼30,000 for both groups), while neutralizing activity was barely detectable in the SARS-CoV-2 S and SARS-CoV-2 S-NP immunized groups (Figure 4B). After 3 immunizations, the titers against SARS-CoV in the cocktail and mosaic S-NP groups did not further improve (median ID_50_ values of 19,936 and 30,210, respectively). The neutralization in the SARS-CoV-2 S and SARS-CoV-2 S-NP groups did improve at week 14 in particular for the SARS-CoV-2 S-NP group (median ID_50_ values of 1,360 versus 9,022, p = 0.0022, respectively), but they were still lower than those in the cocktail and mosaic S-NP groups (median ID_50_ values of 9,022 versus 19,936, p = 0.0043; and versus 30,210, p = 0.0022) (Figure 4B). The mice samples could only be tested against the autologous viruses because of low availability.

SARS-CoV-2 S induced the most potent neutralizing response against SARS-CoV-2 in rabbits after two immunizations, while the SARS-CoV-2 S-NP-induced response was slightly lower (median ID_50_ values of 59,704 and 27,576, p = 0.0556, respectively). The cocktail and mosaic S-NP-induced NAb responses were significantly lower compared with SARS-CoV-2 S (median ID_50_ values of 16,753, p = 0.0159; and 9,540, p = 0.0079, respectively) (Figure 4C). Neutralizing titers against SARS-CoV were potent in both the cocktail and mosaic S-NP groups (medians of 204,174 and 234,423, respectively) while substantially lower in the SARS-CoV-2 S and SARS-CoV-2 S-NP groups (medians of 9,804 and 19,498, respectively). SARS-CoV-2 S induced the most potent neutralizing response against Pangolin GD, while the SARS-CoV-2 S-NP-induced response was slightly lower (median ID_50_ values of 265,155 and 106,958, p = 0.2222, respectively). The cocktail and mosaic S-NP induced NAb responses that were similar to those induced by SARS-CoV-2 S-NP (median ID_50_ values of 123,023 and 65,639, respectively) (Figure 4D). SHC014 neutralization was detected in all immunized animals. The cocktail and mosaic S-NP induced the most potent NAb responses (median ID_50_ values of 16,590 and 14,418, respectively), while SARS-CoV-2 S- and SARS-CoV-2 S-NP-induced responses were significantly lower (median ID_50_ values of 2,146 and 2,645, respectively). Similarly, the cocktail and mosaic S-NP groups induced the most potent NAb responses against WIV1 (median ID_50_ values of 266,808 and 215,654, respectively), while responses in the SARS-CoV-2 S and S-NP groups were significantly lower (median ID_50_ values of 28,143 and 32,778, respectively). NAb titers improved only slightly after the third immunization, and the differences between groups were reduced (Figures 4C and 4D). We note that SARS-CoV-2 S-NP immunized animals developed improved heterologous neutralizing titers compared with the SARS-CoV-2 S immunized animals, but in rabbits, this observation was not statistically significant.

### SARS-CoV-2, cocktail, and mosaic NPs induce broad responses against SARS-CoV-2 VOC

**Figure 5:**
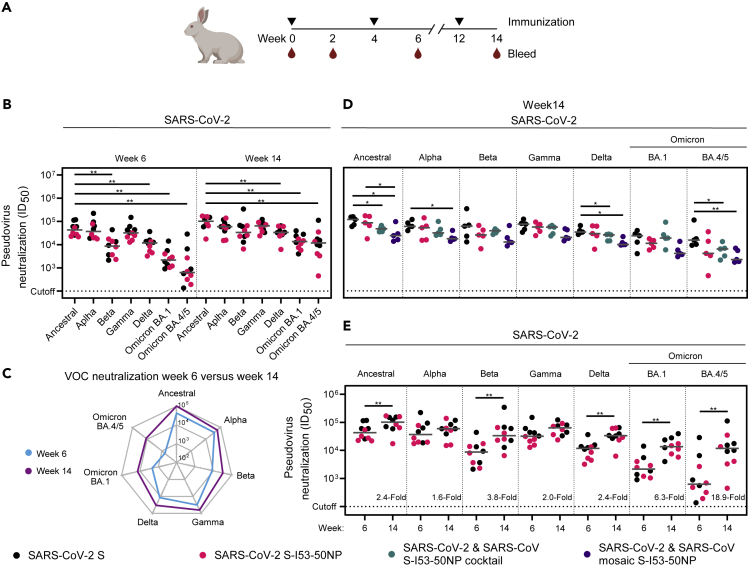
Neutralizing activity against SARS-CoV-2 VOC after SARS-CoV S and SARS-CoV-2 S immunizations in rabbits (A) Study schedule in rabbits. Black triangles indicate immunizations and drops indicate bleeds. (B) SARS-CoV-2 ancestral, Alpha, Beta, Gamma, Delta, and Omicron BA.1 and BA.4/5 pseudovirus neutralization in rabbits at week 6 and 14. SARS-CoV-2 S and SARS-CoV-2 S-NP groups were pooled. (C) SARS-CoV-2 ancestral, Alpha, Beta, Gamma, Delta, and Omicron BA.1 and BA.4/5 pseudovirus neutralization in rabbits at week 6 (blue) and 14 (purple). SARS-CoV-2 S and SARS-CoV-2 S-NP groups were pooled. Lines represent median neutralization titers. (D) SARS-CoV-2 ancestral, Alpha, Beta, Gamma, Delta, and Omicron BA.1 and BA.4/5 pseudovirus neutralization in rabbits for each individual group. Titers between groups were compared using the Mann-Whitney *U* test (∗, p < 0.05; ∗∗, p < 0.01). (E) SARS-CoV-2 ancestral, Alpha, Beta, Gamma, Delta, and Omicron BA.1 and BA.4/5 pseudovirus neutralization, two versus three immunizations, in rabbits. SARS-CoV-2 S and SARS-CoV-2 S-NP groups were pooled. (B, D, and E) The median titers are indicated by a bar. (B and E) Titers between strains and time points were compared using the Wilcoxon test (∗∗, p < 0.01).

The rabbit sera were tested against the SARS-CoV-2 VOC (Figure 5A). SARS-CoV-2 S and SARS-CoV-2 S-NP induced similar levels of NAbs against the VOC, although the extent of neutralization differed per VOC. For a direct comparison between the different VOC, we therefore pooled the animals from the SARS-CoV-2 S and SARS-CoV-2 S-NP groups. At week 6, neutralization of the ancestral strain was the most potent (median ID_50_ value of 42,849) (Figure 5B). Responses against Alpha and Gamma were only slightly lower (median ID_50_ values of 36,908 and 31,999, respectively), while titers against Beta, Delta, and Omicron BA.1 and BA.4/5 were 4.9-, 3.6-, 20-, and 68-fold lower compared with ancestral (medians of 8,684, p = 0.0020 for the difference with the ancestral strain; 11,765, p = 0.0020; 2,158, p = 0.0020; and 626, p = 0.0020, respectively). At week 14, after the third immunization, these differences were reduced and neutralization breath was expanded (Figure 5C). Thus, neutralization titers against Beta, Delta, and Omicron BA.1 and BA.4/5 were 3.0-, 3.0-, 7.5-, and 8.6-fold lower compared with ancestral SARS-CoV-2 (median ID_50_ values of 101,356 versus 33,392, p = 0.1055; and versus 33,419, p = 0.0020; and versus 13,565, p = 0.0020, and versus 11,814, p = 0.0020, respectively) (Figure 5B). Overall, the cocktail and mosaic NPs induced ∼2–3-fold lower levels of ancestral and VOC neutralization compared to the SARS-CoV-2 S and S-NP. The differences were statistically significant in the cases of the ancestral, Alpha, Delta, and Omicron BA.4/5 strains and consistent with the 2-fold reduced quantity of SARS-CoV-2 S in these immunogens (Figure 5D).

The neutralization of VOC increased after a third immunization. Statistically significant titer increases of 2.4-, 3.8-, 2.4-, 6.3-, and 18.9-fold were observed for ancestral, Beta, Delta, and Omicron BA.1 and BA.4/5 in the SARS-CoV-2 S and S-NP groups (median ID_50_ values of 101,356 versus 42,849, p = 0.0059; 33,392 versus 8,684, p = 0.0039; 33,419 versus 11,756, p = 0.0059; 13,565 versus 2,158, p = 0.0020; and 11,814 versus 626, p = 0.0020, respectively). For Alpha and Gamma, we observed an increase of 1.6- and 2.0-fold, but this increase was not statistically significant (median ID_50_ values of 59.198 versus 36.903, p = 0.8457; and 63.810 versus 31.999, p = 0.1602, respectively) (Figure 5E). These findings are consistent with previous studies showing that a third immunization with ancestral S can improve protection against the Beta, Delta, and Omicron variants in particular.^32^^,^^38^^,^^39^

## Discussion

The continuing emergence of SARS-CoV-2 variants poses challenges in controlling the COVID-19 pandemic. While current vaccines, particularly the mRNA vaccines when given multiple times, are still effective at preventing hospitalization and death by SARS-CoV-2 VOC,^40^^,^^41^ their ability to prevent infection by the more recent VOC, i.e., Omicron and its sublineages, is substantially reduced. It is therefore imperative to search for vaccine modalities that increase the breath of protection.

As observed by multiple groups in humans,^42^^,^^43^^,^^44^ ancestral SARS-CoV-2 S induced the lowest NAb titers against Omicron out of all VOC. Neutralizing titers were ∼20- and ∼68-fold lower against Omicron BA.1 and BA.4/5 compared with ancestral SARS-CoV-2 after two immunizations and ∼7.5- and ∼8.6-fold lower after three. Interestingly, out of all VOC, NAb responses against the Omicron variants had the most to gain from a third immunization. We observed a ∼6.3- and ∼18.9-fold increase comparing peak titers after three versus two vaccinations against Omicron BA.1 and BA.4/5, respectively. A third vaccination induces similarly improved neutralizing responses against VOC in humans, in particular against Omicron.^45^^,^^46^ These data reinforce findings that a third vaccination with a Wuhan-Hu-1-based vaccine can significantly improve NAb responses and subsequently protection against Omicron. While vaccine manufacturers have generated booster vaccines specifically targeting SARS-CoV-2 variants, the benefit of such boosters over ones based on the ancestral strain has so far been limited.^21^ A meta-analysis based on neutralization titers from clinical studies comparing ancestral-based and variant-based booster vaccinations predicted that a variant-based booster provides only a modest increase in protection. “A large proportion of the benefit comes from receiving any booster at all (including an ancestral-based booster).”^47^

**Figure 6:**
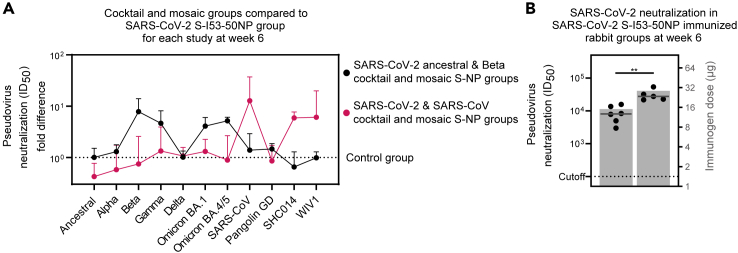
Comparison of SARS-CoV/SARS-CoV-2 and SARS-CoV-2 Wuhan/Beta studies in rabbits (A) Comparison of SARS-CoV-2 ancestral, Alpha, Beta, Gamma, Delta, and Omicron BA.1 and BA.4/5, SARS-CoV, Pangolin GD, SHC014, and WIV1 pseudovirus neutralization in rabbits immunized with SARS-CoV and SARS-CoV-2 cocktail and mosaic S-NP or SARS-CoV-2 ancestral and Beta cocktail and mosaic S-NP to SARS-CoV-2 S-NP groups. Horizontal dotted line represents the neutralization ID_50_ value in the appropriate SARS-CoV-2 S-NP immunized control group for each virus. Fold differences in neutralization ID_50_ compared to the control group are plotted on the y axis. Error bars indicate 95% CI. (B) Comparison of SARS-CoV-2 pseudovirus neutralization in rabbits immunized with 15 μg or 30 μg at week 6. Left y axis shows the neutralization titer, shown as dots in the graph. Right y axis shows immunogen dose, shown as columns in the graph. The median titers are indicated by a bar. Titers between groups were compared using the Mann-Whitney *U* test (∗∗, p < 0.01).

In an attempt to broaden responses against SARS-CoV-2 variants, we immunized rabbits with a combination SARS-CoV-2 ancestral and Beta S presented on NPs. We found that the inclusion of SARS-CoV-2 Beta S improved NAb responses against SARS-CoV-2 Beta, Gamma, and Omicron BA.1 and BA.4/5 by 6.2-, 4.7-, 7.3-, and 4.6-fold compared with immunizations with ancestral SARS-CoV-2 S (Figure 6A). These strains all share mutations in the RBD, including the K417N/T, E484K, and N501Y AA changes. However, we note that the combination of ancestral and Beta S did not outperform immunizations with only Beta S, suggesting that Beta S was responsible for the neutralization breadth against Gamma and Omicron.

Sera from Beta-infected individuals do not quite show the same cross-reactivity as we observe here. Multiple groups have reported cross-neutralizing activity against Gamma, but not Omicron.^43^^,^^44^^,^^48^ Furthermore, mice immunized with Beta S coupled to virosomes developed weaker NAb responses compared with mice immunized with ancestral SARS-CoV-2 S virosomes.^49^ A couple of factors should be considered when comparing these studies. First, our experiments involved rabbits, which make somewhat different antibodies compared to humans and mice.^50^ Second, presentation on I53-50 NPs, as used in this study, might benefit the immunogenicity of Beta S compared with when it is presented on virosomes or on the virus during natural infection.

In a next iteration, we pursued broadening sarbecovirus NAb responses, by immunizing rabbits and mice with a mixture of SARS-CoV-2 and SARS-CoV S presented on NPs in cocktail or mosaic formats. Adding SARS-CoV S to a SARS-CoV-2 S vaccine improved neutralizing responses against SARS-CoV and the bat viruses SHC014 and WIV1 by 12.8-, 5.9-, and 6.1-fold, respectively, compared with immunizations with just SARS-CoV-2 S-NP in rabbits (Figure 6A), confirming a broadening of the NAb response by administering SARS-CoV and SARS-CoV-2 S together. Neutralizing responses against Pangolin GD were not improved by the inclusion of SARS-CoV S in the vaccinations. SHC014 and WIV1 are more closely related to SARS-CoV, while Pangolin GD is more closely related to SARS-CoV-2. This explains why SHC014 and WIV1 neutralizing responses benefit most from the presence of SARS-CoV S, while Pangolin GD neutralizing responses benefit most from the presence of SARS-CoV-2 S. In another study, immunizations of mice with the same I53-50 NPs but displaying a mix of sarbecovirus RBDs, including SARS-CoV-2 and SARS-CoV, induced improved SARS-CoV neutralization compared with SARS-CoV-2 RBD-NPs, but neutralization of SHC014 was not enhanced.^51^ These data suggest that S-NPs may induce stronger cross-reactive responses compared with RBD-NPs, although a direct comparison is impossible because of the different animal models used.

Comparing both our rabbit studies, we observed a 3.4-fold difference in titers against ancestral SARS-CoV-2 in the SARS-CoV-2 S-NP control groups (median titers of 8.073 and 27.576, p = 0.0043) (Figure 6B), which is likely explained by the use of different doses (15 versus 30 μg of SARS-CoV-2 S-NP, respectively). A reduction of SARS-CoV-2 neutralizing activity was also observed in the groups that received combinations of SARS-CoV-2 S and SARS-CoV S, which can probably be explained by the reduction of the amount of SARS-CoV-2 S in the vaccine formulation by 50%. We conclude that dosage of SARS-CoV-2 S in vaccines can have substantial effect on NAb responses, necessitating careful evaluation during vaccine development.

To conclude, we showed that SARS-CoV-2 S proteins from different VOC and SARS-CoV can be co-displayed on self-assembling protein NPs. The I53-50 NP platform has shown to be extremely suitable for co-displaying multiple different proteins due to its two-component nature. The platform is also translatable as an I53-50-based COVID-19 vaccine was recently approved for use in humans in South Korea.^31^ Here, we studied the combination of ancestral and Beta S, while other S combinations might induce broader responses. For instance, one could consider combinations of SARS-CoV-2 Beta and Delta or Delta and Omicron, which carry more distinct mutations. Furthermore, co-displaying a combination of SARS-CoV-2 and SARS-CoV S improved heterologous sarbecoviruses NAb responses. Expanding the panel of sarbecovirus S proteins displayed on NPs provides opportunities to further broaden sarbecovirus immunity. Similar approaches, using RBDs from S proteins of multiple SARS and SARS-like strains, have demonstrated potential in mice and non-human primates.^51^^,^^52^ Future mosaic NP approaches may exclude SARS-CoV-2 S to specifically boost cross-reactive responses. Mosaic NPs that generate pan-sarbecovirus immunity or beyond could offer protection against future SARS-CoV-2 variants or other viruses with pandemic potential.

### Limitations of the study

The data presented here show that combining sarbecovirus S proteins in immunizations can broaden neutralizing responses toward related viruses. There are some limitations to our study that we should note. First, in the SARS-CoV and SARS-CoV-2 S immunization study, we did not include a control group for SARS-CoV S-only immunizations, which could have provided some extra insight into the neutralization breath obtained in the SARS-CoV and SARS-CoV-2 S groups. Second, whether the vaccines tested in this study induce neutralization breath when given as boosters after multiple rounds of ancestral SARS-CoV-2 S immunizations warrants further investigation. Third, the study was performed in mice and rabbits, which are different from humans.

## STAR★Methods

### Key resources table

**Table:** 

REAGENT or RESOURCE	SOURCE	IDENTIFIER
**Antibodies**		

COVA1-18	(Brouwer et al., 2020^7^)	N/A
COVA2-02	(Brouwer et al., 2020^7^)	N/A
COVA2-15	(Brouwer et al., 2020^7^)	N/A
COVA2-39	(Brouwer et al., 2020^7^)	N/A
COVA1-22	(Brouwer et al., 2020^7^)	N/A
Goat anti-rabbit	Jackson Immunoresearch	Cat# 111-035-144; RRID: AB_2307391

**Chemicals, peptides, and recombinant proteins**		

PBS	Thermo Fisher	Cat# 10010023
PEI MAX	Polysciences	Cat# 24765-1
3,3′,5,5′-tetranethylbenzidine	Sigma-Aldrich	Cat# T4444
Squalene Emulsion adjuvant	Polymun Scientific	N/A
Polyinosinic-polycytidylic acid	Invivogen	Cat# vac-pic
Poly-L-Lysine Hydrobromide	Sigma-Aldrich	Cat# P1399
Casein buffer	Thermo Scientific	Cat# 37528
Penicillin	Sigma-Aldrich	Cat# P3032-10MI
Streptomycin	VWR	Cat# 382-EU-100G
SARS-CoV-2 Beta S-I53-50A.1NT1	This Study	N/A
SARS-CoV S-I53-50A.1NT1	This Study	N/A
iRT peptides	Biognosys	Cat# Ki-3002-2
Heavy-labeled peptides	Pepscan	N/A
SDS	Sigma-Aldrich	Cat# L4509
Tris(2-carboxyethyl)phosphine	Sigma-Aldrich	Cat# SBR00051
Chloroacetamide	Sigma-Aldrich	Cat# C0267
Trypsin	Promega	Cat# V5117
LysC	Promega	Cat# VA1170
SARS-CoV peptide 1 DGIYFAATEK	Pepscan	N/A
SARS-CoV peptide 2 AISQIQESLTTTSTALGK	Pepscan	N/A
SARS-CoV-2 peptide 1 VYSTGSNVFQTR	Pepscan	N/A
SARS-CoV-2 peptide 2 VTLADAGFIK	Pepscan	N/A
A-component peptide 1 GPFPNVK	Pepscan	N/A
A-component peptide 2 LFPGEVVGPQFVK	Pepscan	N/A

**Critical commercial assays**		

Nano-Glo Luciferase Assay System	Promega	Cat# N1130
Pierce™ BCA Protein Assay Kit	Thermo Scientific	Cat# 23225

**Experimental models: Cell lines**		

FreeStyle 293F cells	Thermo Fisher	Cat# R79007
HEK 293T/ACE2 cells	(Schmidt et al., 2022^54^)	N/A
HEK 293T cells	ATCC	Cat# CRL-11268

**Experimental models: Organisms/strains**		

BALB/cAnNCrl mice	Charles River Laboratories	N/A
New Zealand White rabbits	Covance Research Products, Inc	N/A

**Recombinant DNA**		

pHIV-1_NL43_ΔENV-NanoLuc plasmid	(Schmidt et al., 2022^54^)	N/A
SARS-CoV-2-S_Δ19_ plasmid	(Schmidt et al., 2022^54^)	N/A
SARS-CoV-2 Alpha-S_Δ19_ plasmid	This study	N/A
SARS-CoV-2 Beta-S_Δ19_ plasmid	This study	N/A
SARS-CoV-2 Gamma-S_Δ19_ plasmid	This study	N/A
SARS-CoV-2 Delta-S_Δ19_ plasmid	This study	N/A
SARS-CoV-2 Omicron BA.1-S_Δ19_ plasmid	This study	N/A
SARS-CoV-2 Omicron BA.4/5-S_Δ19_ plasmid	This study	N/A
SARS-CoV-2 SARS-CoV-S_Δ19_ plasmid	This study	N/A
SARS-CoV-2 Pangolin GD-S_Δ19_ plasmid	This study	N/A
SARS-CoV-2 SHC014-S_Δ19_ plasmid	This study	N/A
SARS-CoV-2 WIV1-S_Δ19_ plasmid	This study	N/A
SARS-CoV-2 Beta S-I53-50A.1NT1 pPPI4 plasmid	This study	N/A
SARS-CoV S-I53-50A.1NT1 pPPI4 plasmid	This study	N/A
SARS-CoV-2 S-dn5 pPPI4 plasmid	(Brouwer et al., 2021^25^)	N/A
SARS-CoV S-dn5 pPPI4 plasmid	(Brouwer et al., 2021^25^)	N/A

**Software and algorithms**		

GraphPad Prism v8	GraphPad	N/A
UCSF ChimeraX	(Goddard et al., 2018^55^)	N/A
Adobe Illustrator	Adobe	N/A
Skyline Daily Version 22.1.9.208	(Pino et al., 2017^56^)	https://skyline.ms
R version 4.1.3	R	https://www.r-project.org/
RStudio version 2022.07.1 + 554	RStudio	https://rstudio.com/

**Other**		

Ni-NTA agarose	QIAGEN	Cat# 30210
Ni-NTA HighSorb plates	QIAGEN	Cat# 35061
Superose 6 increase 10/300 GL	Sigma-Aldrich	Cat# GE29-0915-96
Econo-column chromatography columns	BIO RAD	Cat# 7371512
NGC chromatography system	BIO RAD	N/A
Octet K2 system	Sartorius (FortéBio)	N/A
Octet Biosensors: Protein A	Sartorius (FortéBio)	Cat# 18-5010
Vivaspin 20, 100.000 kDa MWCO, Polyethersulfone	Sigma-Aldrich	Cat# GE28-9323-63
Nucleobond Xtra Maxi kit	Macherey-Nagel	Cat# 740414.50
Fast Digest BamHI	Thermo Scientific	Cat# FD0054
Fast Digest Green buffer 10x	Thermo Scientific	Cat# B72
Fast Digest PstI	Thermo Scientific	Cat# FD0614
FreeStyle 293 Expression medium	Thermo Scientific	Cat# 12338018
DMEM	Sigma-Aldrich	Cat# D6429-500ML
Glutamax supplement	Thermo Fisher	Cat# 35050061
High-binding plates: Half-area 96-well polystyrene high-binding microplate	Greiner	Cat# 675061
Steritop Filter Units	Merckmillipore	Cat# C3239
Glomax	Turner BioSystems	Model# 9101-002
Microplate 96 well half area white	Greiner bio-one	Cat# 675074
Greiner CELLSTAR® 96 well plates round bottom clear wells	Merck	Cat# M9436
AKTA Avant150 FPLC system	Cytiva	N/A
Protein A Magnetic Beads	ThermoFisher	Cat# 88845
S-trap micro	Protifi	Cat# C02-micro-80

### Resource availability

#### Lead contact

Further information and requests for resources and reagents should be directed to and will be fulfilled by the lead contact, Rogier W. Sanders (r.w.sanders@amsterdamumc.nl).

#### Materials availability

All reagents will be made available on request after completion of a Materials Transfer Agreement.

### Experimental model and subject details

#### Cell lines

HEK 293T (ATCC CRL-11268) and HEK 293F (Life Technologies) are female human embryonic kidney cell lines transformed for increased production of retrovirus or recombinant protein. HEK 293F cells are adapted to grow in suspension. HEK 293T cells were cultured in flasks with DMEM +10% FBS +1% penicillin-streptomycin at 37°C with 5% CO_2_. HEK 293F cells were cultured in 293FreeStyle expression medium (Life Technologies) at 37°C with 8% CO_2_ and shaking at 125 rpm. HEK 293T/ACE2 is a human embryonic kidney cell line expressing Human Angiotensin-Converting Enzyme 2. HEK 293T/ACE2 cells were cultured in flasks with DMEM +10% FBS +1% penicillin-streptomycin at 37°C with 5% CO_2_.

#### Rabbits

Female New Zealand White rabbits, aged ∼6 months, from multiple litters of 2.5–5 kg were used in this study. The animals were sourced and housed at Covance Research Products, Inc. (Denver, PA, USA). Immunizations were performed under permits with approval number C0079–21 and C0084-20. Immunization procedures complied with protocols of the Covance Institutional Animal Care and Use Committee and all relevant ethical regulations.

#### Mice

Female BALB/cAnNCrl mice, aged 8 weeks, were ordered from Charles River Laboratories and housed at the Animal Research Institute Amsterdam under BSL-2 conditions. All experiments were performed in accordance with the Dutch Experiment on Animals Act and were approved by the Animal Ethics Committee of the Amsterdam UMC (Permit number 17-4045).

### Method details

#### Construct design

The SARS-CoV-2-S-I53-50A.1NT1 plasmid was described before.^25^ In short, the previously described pPPI4 plasmid encoding the prefusion SARS-CoV-2 S protein^7^ was digested with PstI and BamHI and ligated in a PstI-BamHI-digested pPPI4 plasmid encoding a modified I53-50A.1NT1 sequence. The original I53-50A.1NT1 plasmid was described previously.^30^ Modifications constitute the introduction of GSLEHHHHHH after the final residue to introduce a C-terminal histidine-tag. To create the SARS-CoV-2-S-Beta-I53-50A.1NT1, the above plasmid was digested with PstI and BamHI to generate the plasmid backbone and a Gblock (Integrated DNA Technologies) encoding the prefusion SARS-CoV-2 Beta S protein was cloned into the backbone using Gibson Assembly. To create the prefusion SARS-CoV-2 Beta S the following mutations were included in the SARS-CoV-2 S sequence: L18F, D80A, D215G, L242H, R246I, K417N, E484K, N501Y, D614G and A701V. The SARS-CoV-S-I53-50A.1NT1 was created using the same backbone and cloning in a Gblock encoding prefusion SARS-CoV S protein with Gibson Assembly. The prefusion SARS-CoV S construct was designed as described before.^57^ Prefusion SARS-CoV-2 S and SARS-CoV S genes were also cloned into pPPI4 plasmid encoding dn5 trimerization domain^58^ to generate S-dn5 fusion proteins to be used in ELISAs. All protein constructs contained a C-terminal His_6_-tag to facilitate purification as well as oriented immobilization in ELISA experiments.

#### Protein expression and purification

All constructs were transiently transfected into HEK 293F cells (Invitrogen) maintained in Freestyle medium (Life Technologies) at 0.8–1.2 million cells/mL. For transfection, a mix of expression plasmid (312.5 μg/L cells) and PEImax (937.5 μg/L cells) was made in OptiMEM (Gibco) and was added to the cells. Six days after transfection, supernatants were collected by centrifuging cell cultures at 3000 × *g* for 30 min. Supernatants were filtered using 0.22 μm Steritop filters (Merck Millipore) and subjected to Ni-NTA agarose beads for affinity purification. Eluted proteins were concentrated and buffer exchanged to PBS using Vivaspin (GE Healthcare) filters with a 100.000 Da cutoff. Protein concentrations were measured using Nanodrop using the proteins peptidic molecular weight.

#### S-I53-50 NP assembly

S-I53-50A.1NT1 fusion proteins were buffer exchanged into TBS and sterile filtered using a 0.22 μm spin column. Proteins were applied to a Superose 6 increase 10/300 GL column (GE healthcare) in TBS with 5% glycerol. Appropriate size-exclusion fractions were collected and pooled. Equimolar amounts of I53-50B.4PT1 were mixed with the S-I53-50A.1NT1 sample and incubated at 4°C overnight. For the assembly of mosaic I53-50 NPs, the appropriate S-I53-50A.1NT1 were mixed at equimolar amounts before adding I53-50B.4PT1. After overnight assembly, particles were applied to a Superose 6 increase 10/300 GL column in TBS+5% glycerol to remove unassembled components. Appropriate size-exclusion fractions were collected and concentrated using a 10.000 Da Vivaspin column (GE healthcare). Protein concentrations were measured using Nanodrop using the proteins peptidic molecular weight.

#### Negative-stain EM

S-I53-50 NPs were added to carbon-covered 400 mesh copper grids and stained with 2% uranyl formate. Micrographs were imaged on a Tecnai F12 Spirit microscope with a 4k FEI Eagle CCD. Leginon^59^ and Appio^60^ were used to collect and process micrographs.

#### BLI assay

S-I53-50A.1NT1 and SARS-CoV-2 S-I53-50 NP samples were diluted to 100 nM and 5 nM, respectively, in PBS with 0.1% bovine serum albumin and 0.02% Tween 20, and antibody binding was assessed using a ForteBio Octet K2. Assays were performed at 30°C with agitation set at 1000 rpm. Antibodies were loaded on protein A sensors (ForteBio) at 10 μg/mL in PBS with 0.1% bovine serum albumin and 0.02% Tween 20 until a binding threshold of 1 nm was reached. Association and dissociation were measured for 300 s.

#### Immunoprecipitation

Magnetic Dynabeads Protein A (Invitrogen) were washed with PBS using a magnetic tube rack. Beads were incubated with COVA-18 at 0.7 μg antibody per 1 μL beads for 2 h at room temperature in an overhead rotator. Excess antibodies were removed by washing the beads with PBS. One μg of SARS-CoV SARS-CoV-2 mosaic I53-50 NP was added for every μg of antibody and the mixture was incubated at room temperature in an overhead rotator overnight. Beads were washed with PBS to remove unbound particles and protein was eluted by boiling for 10 min in PBS.

#### Spectral library generation

First, spectral libraries were generated to determine peptide fragmentation characteristics and retention times. Digested spike trimers were mixed with iRT peptides (Biognosys) and measured using a Q-Exactive HF (Thermo Scientific). An unscheduled parallel reaction monitoring (PRM) method was used that scanned for the +2 and +3 charged peptides. Peptides were separated using a 65 min gradient and a 120 k resolution was used for the PRM assay, resulting in a minimum of 5 spectra per peptide. Raw files were analyzed using Byonic (version 4.1.10), carbamidomethyl using cysteine as fixed modification, and the variable modifications serine/threonine/tyrosine phosphorylation, methionine oxidation, and deamidation. The search results were filtered using a 1% FDR cut off, and manually validated for quality. Subsequently using Skyline Daily (version 20.2.1.404) pseudo-MS2 spectra were generated which were used as the peptide library. The custom mix of heavy labeled peptides was subsequently ordered and compared to the spectral library (Pepscan, Lelystad, the Netherlands).

#### SRM assay development

The SRM assay was developed using previous described methods.^61^ The assay was developed on a TSQ Altis (Thermo Scientific). In brief, the 10 most intense fragment ions from the library were used as initial transitions. These transitions were used to optimize multiple parameters such as retention time and collision energy. Collision energy was optimized per transition using Skyline, with the TSQ Vantage CE formula as starting point (CE = 0.03 *m*/*z* + 2.905 for doubly charged precursors and CE = 0.038 *m*/*z* + 2.281 for precursor charges of three and higher) and optimized using steps of 1 voltage.

#### Quantification sample preparation

Sample preparation of the nanoparticles was performed following the suppliers’ protocol S-Trap (Protifi) with small adjustments. In short, 2 μg of nanoparticle was dissolved in 5% SDS and 50 mM TRIS at pH 8.5. Samples were reduced for 30 min at 60°C in 5mM tris(2-carboxyethyl)phosphine (TCEP), alkylated in dark for 30 min using 20 mM chloroacetamide (CAA), and quenched using TCEP. Phosphoric acid was added to a final concentration of ∼2.5%, following regular protocol loading into the S-trap column and incubated overnight at 37°C with 1μg of trypsin. Peptides were eluted using 40 μL of 50mM TRIS pH 8.5 containing 100fmol of the heavy labeled synthetic peptides, 40 μL 0.2% formic acid, and 40 μL 50% acetonitrile. Samples were subsequently dried down and subject to SRM analysis.

#### SRM LC–MS/MS setup

Samples were analyzed on a TSQ Altis (Thermo Scientific) coupled to an UltiMate 3000 (Thermo Scientific), and an easy spray analytical column (ES802A, 25 cm, 75 mm ID PepMap RLSC, C18, 100 A˚, 2 mm particle size column (Thermo Scientific)). First, samples were reconstituted in 2% LC–MS grade formic acid, containing the heavy labeled peptides. Samples were loaded on a trap column (Acclaim™ PepMap™ 100C18 HPLC Column 0.3 × 5mm with 5 μm particles (Thermo Scientific)) with 2.2% Buffer A (0.1% FA) for 3 min, and subsequently separated using 0-32% buffer B (99.9%ACN, 0.1%FA) in 35 min at 300 nL/min. Followed by a 20 min column wash with 80% buffer B at 300 nL/min, and 10 min column equilibration at 2.2% B. The TSQ Altis spray voltage was set at 1.9 kV and fragmented at 1.5 mTorr in the second quadrupole. The first quadrupole was set at 0.7 da FWHM, and the third quadrupole at 1.2 da FWHM. All transitions were measured with an optimized collision energy without scheduling and a cycle time of 1.5 s.

#### SRM data assessment

All experiments were analyzed using Skyline Daily (version 20.2.1.404). Quality of the peptides was assessed mainly on the signal similarity between the heavy and the light peptides. Most important aspects were perfect co-elution, peak shape, and relative contributions of each transition between the heavy and the light peptide. A rdotp >0.95 was maintained as an indicator of the similarity between the heavy and the light peptide. Normalization, quantification analysis, and Gaussion distribution calculation of the population heterogeneity was done using in-house R (version 4.1.1) scripts.

#### Animals and study designs

##### SARS-CoV-2 ancestral and Beta study

Six female New Zealand White rabbits per group were given two intramuscular immunizations, one in each quadricep, at weeks 0 and 4. Animals were immunized with SARS-CoV-2 S-I53-50 NP, a cocktail of SARS-CoV-2 S-I53-50 NP and SARS-CoV-2 Beta S-I53-50 NP or SARS-CoV-2 Wuhan & Beta mosaic S-I53-50 NP formulated 1:1 in Squalene Emulsion adjuvant (Polymun, Klosterneuburg, Austria). Animals were immunized with 15 μg of S protein on I53-50 NP. Rabbits were bled at week 0, 4 and 6.

##### SARS-CoV and SARS-CoV-2 study

Eight twelve week old female Balb/c mice per group received subcutaneous immunizations into the neck skin-fold at weeks 0, 4, and 12. Animals were immunized with a cocktail of SARS-CoV S-I53-50 NP and SARS-CoV-2 S-I53-50 NP or SARS-CoV & SARS-CoV-2 mosaic S-I53-50 NP adjuvanted with 50 μg of polyinosinic-polycytidylic acid (Poly-IC; Invivogen). Animals were immunized with 10 μg of S protein on I53-50 NP. Blood was collected at weeks −1, 2, 6 and 14. Two out of eight mice were sacrificed at week 6.

Five female New Zealand White rabbits per group were given two intramuscular immunizations, one in each quadricep, at weeks 0, 4 and 12. Animals were immunized with a cocktail of SARS-CoV S-I53-50 NP and SARS-CoV-2 S-I53-50 NP or SARS-CoV & SARS-CoV-2 mosaic S-I53-50 NP formulated 1:1 in Squalene Emulsion adjuvant (Polymun, Klosterneuburg, Austria). Animals were immunized with 30 μg of S protein on I53-50NP. Rabbits were bled at week 0, 2, 6 and 14.

#### Depletion ELISA

Ni-NTA HighSorb plates were coated with 2 μg/mL SARS-CoV S-dn5 or SARS-CoV-2 S-dn5 in TBS for 2h. After coating, plates were blocked with TBS containing 2% milk for 1 h. Serum depletion was done in a separate 96-well plate. In this plate, TBS containing 2% milk and 20% sheep serum was added to row 2-8 and depletion reagents SARS-CoV S-dn5, SARS-CoV-2 dn5 (all at 5 μg/mL) or PBS were added to individual columns. Serum dilutions in TBS containing 2% milk and 20% sheep serum were added to the first row of the plate and the depletion reagent was added. Serial dilutions were made until row 7 and incubated for 1 h. The ELISA plate was washed two times with TBS and the depleted serum samples were added and incubated for 2h. Plates were washed two times with TBS and a secondary HRP-labeled Goat-*anti*-Rabbit antibody in TBS with 2% milk at 1:500 was added to the wells and incubated for 1h. Plates were then washed five times with TBS containing 0.05% tween-20. Developing solution (1% 3,3′,5,5′-tetranethylbenzidine (Sigma-Aldrich), 0.01% H_2_O_2_, 100 mM sodium acetate and 100 mM citric acid) was added to each well and the developing reaction was stopped by adding 0.8 M H_2_SO_4_ after 1 min. Luminescence was measured at and OD of 450 and midpoint titers were determined. IC_50_s were used to determine the extent of depletion, no depletion or complete depletion (i.e. the equivalent depletion as with the homologous protein) were set at 100 and 0%, respectively, and the decrease in binding with the heterologous spike (%) was calculated accordingly.

#### Pseudovirus neutralization assay

Neutralization assays and the generation of a SARS-CoV-2, SARS-CoV, Pangolin GD, SHC014 and WIV1 pseudovirus containing a NanoLuc luciferase reporter gene were performed as described elsewhere.^54^ For production of pseudovirus, HEK 293T cells (ATCC, CRL-11268), grown in DMEM (Gibco), supplemented with 10% fetal bovine serum (FBS), penicillin (100 U/mL), and streptomycin (100 μg/mL), were transfected with a pHIV-1_NL43_ΔENV-NanoLuc reporter virus plasmid and a SARS-CoV-S_Δ19_, SARS-CoV-2-S_Δ19_, SARS-CoV-2-Alpha-S_Δ19_, SARS-CoV-2-Beta-S_Δ19_, SARS-CoV-2-Gamma-S_Δ19_, SARS-CoV-2-Delta-S_Δ19_, SARS-CoV-2-Omicron-BA.1-S_Δ19_, SARS-CoV-2-Omicron-BA.4/5-S_Δ19_, Pangolin GD-S_Δ19_,SHC014-CoV-S_Δ19_ or WIV1-S_Δ19_ expression plasmid. 48 h after transfection, cells supernatants were harvested, centrifuged for 5 min at 500 × *g* and sterile filtered through a 0.22 μm pore size PVDF syringe filter. For the neutralization assays, HEK 293T expressing the SARS-CoV-2 receptor ACE2 (HEK 293T/ACE2) were cultured in the same medium as HEK 293T. To determine the neutralization activity in serum samples, HEK 293T/ACE2 cells were first seeded in 96-well plates coated with 50 μg/mL poly-l-lysine at a density of 2 × 10^4^/well in the culture medium described above but with GlutaMax (Gibco) added. The next day, duplicate serial dilutions of heat inactivated serum samples were prepared in the same medium as used for seeding of cells and mixed 1:1 with pseudovirus. This mixture was incubated at 37°C for 1 h before adding it to the HEK 293T/ACE2 cells in a 1:1 ratio with the cell culture medium. After 48 h, the cells were lysed and lysate was transferred into half area 96-well white microplates (Greiner bio-one). Luciferase activity was measured in the lysates using the Nano-Glo Luciferase Assay System (Promega) with a Glomax system (Turner BioSystems). Relative luminescence units (RLU) were normalized to those from cells infected with pseudovirus in the absence of serum. Neutralization titers (ID_50_-titers) were determined as the serum dilution at which infectivity was inhibited by 50%.

### Quantification and statistical analysis

Midpoint binding titers and midpoint neutralization titers were determined using Graphpad Prism 8.0. Comparisons between two experimental groups were made using a Mann-Whitney *U* test and comparisons between time points were made using the Wilcoxon test (∗, p < 0.05; ∗∗, p < 0.01; ∗∗∗, p < 0.001).

## Data Availability

• The data supporting the findings of the study are available from the corresponding author upon reasonable request. • This paper does not report original code. • Any additional information required to reanalyze the data reported in the paper is available from the lead contact upon request.
