# Supplementary material for: Co-display of diverse spike proteins on nanoparticles broadens sarbecovirus neutralizing antibody responses
Source: iScience. 2022 Nov 22;25(12):105649. doi: 10.1016/j.isci.2022.105649 (PMC9678814; doi:10.1016/j.isci.2022.105649)
Supplement: Document S1. Figures S1 and Tables S1–S3 [file mmc1.pdf]

iScience, Volume *25*

Supplemental information

Co-display of diverse spike proteins on nanoparticles broadens sarbecovirus neutralizing antibody responses

Mitch Brinkkemper, Tim S. Veth, Philip J.M. Brouwer, Hannah Turner, Meliawati Poniman, Judith A. Burger, Joey H. Bouhuijs, Wouter Olijhoek, Ilja Bontjer, Jonne L. Snitselaar, Tom G. Caniels, Cynthia A. van der Linden, Rashmi Ravichandran, Julien Villaudy, Yme U. van der Velden, Kwinten Sliepen, Marit J. van Gils, Andrew B. Ward, Neil P. King, Albert J.R. Heck, and Rogier W. Sanders

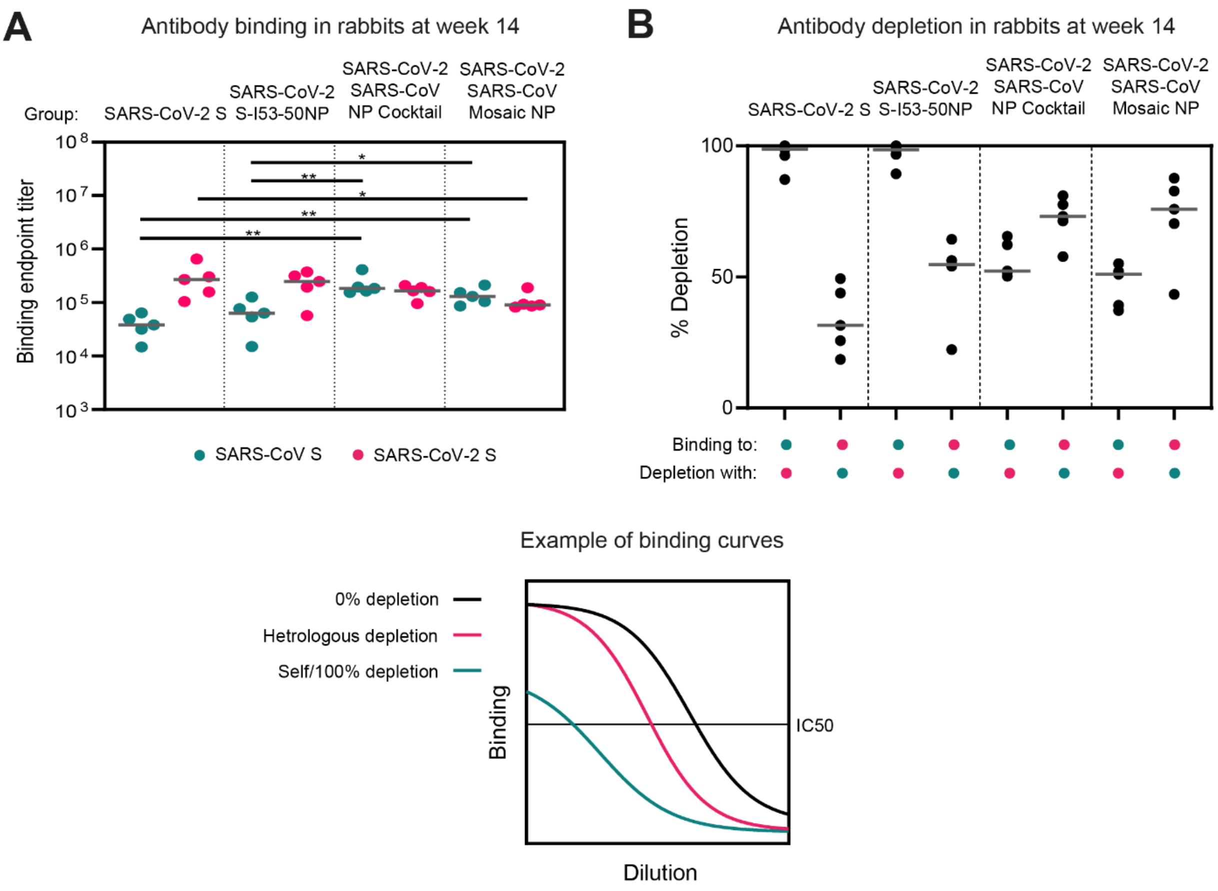

Figure S1. Antibody binding and depletion after SARS-CoV-2 and SARS-CoV S immunizations in rabbits, related to figure 4.

(A) Antibody binding to SARS-CoV and SARS-CoV-2 S at week 14 in rabbits. Titers between groups were compared using the Mann-Whitney U test (*, $p < 0.05$; **, $p < 0.01$). (B) Antibody depletion ELISA with sera from rabbits obtained at week 14. The sera were depleted with SARS-CoV or SARS-CoV-2 S and antibody binding against the heterologous S protein was reported. The graph depicts the loss of binding after depletion calculated by using the difference in IC_{50} in the absence or presence of a depleting agent and calibrated using the homologous protein. An example of binding curves before and after depletion is given on the bottom. Bars indicate the median.

Table S1. SARS-CoV, SARS-CoV-2, Pangolin GD, SHC014 and WIV1 pseudovirus neutralization titers in rabbits immunized with SARS-CoV-2 S Wuhan and Beta, related to figure 2.

	Group	SARS-CoV-2-S						SARS-CoV-2-S I53-50NP						SARS-CoV-2-S I53-50NP SARS-CoV-2-S Beta I53-50NP cocktail						SARS-CoV-2-S Wuhan & Beta mosaic I53-50NP					
	Animal ID	1	2	3	4	5	6	7	8	9	10	11	12	13	14	15	16	17	18	19	20	21	22	23	24
	Virus																								
Week 0	Ancestral	<100	<100	<100	<100	<100	<100	<100	<100	<100	<100	<100	<100	<100	<100	<100	<100	<100	<100	<100	<100	<100	<100	<100	<100
	Alpha	<100	<100	<100	<100	<100	<100	<100	<100	<100	<100	<100	<100	<100	<100	<100	<100	<100	<100	<100	<100	<100	<100	<100	<100
	Beta	<100	<100	<100	<100	<100	<100	<100	<100	<100	<100	<100	<100	<100	<100	<100	<100	<100	<100	<100	<100	<100	<100	<100	<100
	Gamma	<100	<100	<100	<100	<100	<100	<100	<100	<100	<100	<100	<100	<100	<100	<100	<100	<100	<100	<100	<100	<100	<100	<100	<100
	Delta	<100	<100	<100	<100	<100	<100	<100	<100	<100	<100	<100	<100	<100	<100	<100	<100	<100	<100	<100	<100	<100	<100	<100	<100
	Omicron BA.1	<100	<100	<100	<100	<100	<100	<100	<100	<100	<100	<100	<100	<100	<100	<100	<100	<100	<100	<100	<100	<100	<100	<100	<100
	Omicron BA.4/5	<100	<100	<100	<100	<100	<100	<100	<100	<100	<100	<100	<100	<100	<100	<100	<100	<100	<100	<100	<100	<100	<100	<100	<100
	SARS-CoV	<100	<100	<100	<100	<100	<100	<100	<100	<100	<100	<100	<100	<100	<100	<100	<100	<100	<100	<100	<100	<100	<100	<100	<100
	Pangolin GD	<100	<100	<100	<100	<100	<100	<100	<100	<100	<100	<100	<100	<100	<100	<100	<100	<100	<100	<100	<100	<100	<100	<100	<100
	SHC014	<100	<100	<100	<100	<100	<100	<100	<100	<100	<100	<100	<100	<100	<100	<100	<100	<100	<100	<100	<100	<100	<100	<100	<100
	WIV1	<100	<100	<100	<100	<100	<100	<100	<100	<100	<100	<100	<100	<100	<100	<100	<100	<100	<100	<100	<100	<100	<100	<100	<100
Week 4	Ancestral	969	214	358	<100	193	<100	759	727	753	947	192	267	985	880	1076	189	261	<100	774	478	1694	<100	1031	244
	Alpha	154	<100	<100	<100	<100	<100	351	521	784	212	<100	<100	181	392	837	<100	194	<100	202	168	196	<100	279	<100
	Beta	<100	<100	<100	<100	<100	<100	789	1582	498	1063	205	471	591	706	3000	831	656	550	524	610	2973	281	1007	188
	Gamma	<100	<100	<100	<100	<100	<100	947	717	490	613	154	231	218	559	967	119	175	114	731	476	1346	202	777	195
	Delta	<100	<100	<100	<100	<100	<100	239	163	121	163	<100	<100	261	101	382	<100	<100	<100	198	184	248	<100	167	<100
	Omicron BA.1	<100	<100	<100	<100	<100	<100	<100	<100	<100	412	<100	<100	338	<100	189	<100	<100	<100	<100	253	<100	<100	<100	<100
	Omicron BA.4/5	<100	<100	120	<100	<100	<100	121	<100	<100	123	<100	<100	167	<100	312	<100	<100	<100	<100	125	<100	<100	<100	<100
	SARS-CoV	<100	<100	136	<100	<100	<100	163	<100	151	175	<100	<100	145	104	<100	<100	<100	<100	<100	126	<100	<100	136	<100
	Pangolin GD	2086	1154	2091	101	552	367	8253	1948	2039	6185	930	587	1352	3187	4692	2374	2312	1006	1703	2714	2325	990	2560	770
	SHC014	122	148	<100	<100	<100	<100	218	347	350	436	<100	<100	167	417	222	122	<100	<100	276	337	442	181	760	<100
	WIV1	190	162	237	<100	<100	<100	340	116	119	504	<100	<100	294	462	203	<100	206	138	158	602	750	<100	477	126
Week 6	Ancestral	8340	13621	15772	2972	7806	4981	8582	9763	5384	10450	2301	7302	3866	14925	13208	5578	11092	3012	7640	5494	13189	4383	13129	7524
	Alpha	2644	6672	3533	1673	3595	2521	6338	5472	6021	5960	2517	3388	2317	3351	7581	2960	7062	2485	4400	4844	4964	2240	4411	3158
	Beta	2726	4267	2364	480	1052	1410	16350	13613	9678	21885	3439	9113	13703	11867	20178	6703	19265	10009	14279	9681	10914	5154	30686	12386
	Gamma	4578	4615	3438	820	4077	2158	29612	22197	8838	31504	9058	13251	7322	11875	40800	8559	22046	9939	16020	9542	15284	6671	19082	11324
	Delta	3696	7857	10717	2645	6731	3467	6514	4668	3832	6407	1238	5082	2621	12037	8708	2641	8999	3189	4036	3566	8901	2722	8881	5088
	Omicron BA.1	710	598	1379	241	1591	1182	15292	11669	1967	10731	583	2963	3326	9583	7884	1393	3367	1683	6397	3606	1797	837	4566	834
	Omicron BA.4/5	303	488	1124	<100	267	316	7357	1167	993	1960	241	1701	1492	1017	6651	541	4343	1721	910	695	760	453	5044	1915
	SARS-CoV	3627	5153	3707	2290	2881	3051	4842	1265	3242	6119	1292	2180	4291	7714	3278	2922	8491	3342	4410	9052	8150	1929	10542	8884
	Pangolin GD	40618	53744	64352	18243	22030	16963	26997	71871	37367	62457	47841	47803	15984	65383	111152	59239	39832	23054	25657	39818	80071	28602	41022	41704
	SHC014	7793	10631	4277	10129	6816	4356	8130	3100	11191	9808	1324	189	3825	6438	3020	2494	4772	2219	10237	5378	21735	5999	8744	5472
	WIV1	7123	8237	3073	3828	6356	6342	10581	3030	5321	9461	4542	3579	5817	8735	15435	4033	7977	3193	3238	5801	10709	2849	5806	8168

<100
101-1,000
1,001-10,000
10,001-100,000
100,001-1,000,000

Table S2. SARS-CoV and SARS-CoV-2 pseudovirus neutralization titers in mice immunized with SARS-CoV S and SARS-CoV-2 S, related to figure 4.

	Group	SARS-CoV-2-S								SARS-CoV-2-S I53-50NP							
	Animal ID	1	2	3	4	5	6	7	8	9	10	11	12	13	14	15	16
	Virus																
Week -1	Ancestral	<100		<100		<100		<100		<100		<100		<100		<100	
	SARS-CoV	<900		<900		<900		<900		<900		<900		<900		<900	
Week 2	Ancestral	<100	<100	<100	<100	141	<100	<100	<100	371	<100	<100	355	398	724	1000	831
	SARS-CoV	<900	<900	<900	<900	<900	<900	<900	<900	<900	<900	<900	<900	<900	<900	<900	<900
Week 6	Ancestral	2288	5734	2703	2507	7106	12379	4765	32285	75063	25593	2588	23399	2992	8179	14035	19548
	SARS-CoV	<900	<900	<900	<900	<900	<900	<900	<900	<900	3162	<900	<900	<900	2951	<900	<900
Week 14	Ancestral	48827	n.d.	32400	51072	54264	n.d.	25037	36230	n.d.	63015	231100	63418	n.d.	17235	35046	18749
	SARS-CoV	2239	n.d.	<900	1820	<900	n.d.	<900	1905	n.d.	7964	5623	10594	n.d.	10080	3090	11019

	Group	SARS-CoV-S I53-50NP SARS-CoV-2-S I53-50NP cocktail								SARS-CoV-S SARS-CoV-2-S mosaic I53-50NP							
	Animal ID	17	18	19	20	21	22	23	24	25	26	27	28	29	30	31	32
	Virus																
Week -1	Ancestral	<100		<100		<100		<100		<100		<100		<100		<100	
	SARS-CoV	<900		<900		<900		<900		<900		<900		<900		<900	
Week 2	Ancestral	<100	<100	<100	<1000	269	<100	182	1202	<100	<100	602	<100	138	275	209	<100
	SARS-CoV	2089	3388	n.d.	1820	<900	<900	1514	<900	3981	<900	6457	1047	<900	1905	1549	<900
Week 6	Ancestral	39851	541,7	35537	6297	12770	6256	5528	4112	2498	2061	26748	1067	5481	7920	23447	9063
	SARS-CoV	n.d.	12025	30741	30327	11797	25596	95467	36580	21828	28124	55263	20852	19577	30865	59928	42970
Week 14	Ancestral	14588	6099	13489	n.d.	20437	48824	n.d.	n.d.	54228	19812	n.d.	27528	18163	31890	n.d.	58070
	SARS-CoV	142015	14535	50276	n.d.	16993	19936	n.d.	n.d.	94422	23083	n.d.	37084	59541	23336	n.d.	18485

Colour	Range
(green)	<100/<900
(yellow)	101-1,000
(orange)	1,001-10,000
(red)	10,001-100,000
(magenta)	100,001-1,000,000

Table S3. SARS-CoV, SARS-CoV-2, Pangolin GD, SHC014 and WIV1 pseudovirus neutralization titers in rabbits immunized with SARS-CoV S and SARS-CoV-2 S, related to figure 4 and 5.

	Group	SARS-CoV-2-S					SARS-CoV-2-S I53-50NP					SARS-CoV-S I53-50NP SARS-CoV-2-S I53-50NP cocktai					SARS-CoV-S SARS-CoV-2-S mosaic I53-50NP				
	Animal ID	1	2	3	4	5	6	7	8	9	10	11	12	13	14	15	16	17	18	19	20
	Virus																				
Week 0	Ancestral	<100	<100	<100	<100	<100	<100	<100	<100	<100	<100	<100	<100	<100	<100	<100	<100	<100	<100	<100	<100
	Alpha	<100	<100	<100	<100	<100	<100	<100	<100	<100	<100	<100	<100	<100	<100	<100	<100	<100	<100	<100	<100
	Beta	<100	<100	<100	<100	<100	<100	<100	<100	<100	<100	<100	<100	<100	<100	<100	<100	<100	<100	<100	<100
	Gamma	<100	<100	<100	<100	<100	<100	<100	<100	<100	<100	<100	<100	<100	<100	<100	<100	<100	<100	<100	<100
	Delta	<100	<100	<100	<100	<100	<100	<100	<100	<100	<100	<100	<100	<100	<100	<100	<100	<100	<100	<100	<100
	Omicron BA.1	<100	<100	<100	<100	<100	<100	<100	<100	<100	<100	<100	<100	<100	<100	<100	<100	<100	<100	<100	<100
	Omicron BA.4/5	<100	<100	<100	<100	<100	<100	<100	<100	<100	<100	<100	<100	<100	<100	<100	<100	<100	<100	<100	<100
	SARS-CoV	<100	<100	<100	<100	<100	<100	<100	<100	<100	<100	<100	<100	<100	<100	<100	<100	<100	<100	<100	<100
	Pangolin GD	<100	<100	<100	<100	<100	<100	<100	<100	<100	<100	<100	<100	<100	<100	<100	<100	<100	<100	<100	<100
	SHC014	<100	<100	<100	<100	<100	<100	<100	<100	<100	<100	<100	<100	<100	<100	<100	<100	<100	<100	<100	<100
	WIV1	<100	<100	<100	<100	<100	<100	<100	<100	<100	<100	<100	<100	<100	<100	<100	<100	<100	<100	<100	<100
Week 2	Ancestral	748	498	1293	121	476	<100	<100	<100	<100	113	<100	<100	<100	<100	<100	<100	<100	<100	<100	<100
	Alpha	1111	315	1002	<100	1612	<100	<100	102	164	589	121	<100	485	138	228	<100	529	<100	<100	131
	Beta	<100	<100	<100	<100	<100	<100	<100	<100	<100	<100	<100	<100	<100	<100	<100	<100	129	<100	<100	<100
	Gamma	819	1102	1068	<100	317	285	505	1462	579	794	337	451	539	814	503	<100	397	<100	823	168
	Delta	235	149	394	<100	332	<100	<100	<100	<100	<100	<100	<100	<100	<100	<100	<100	<100	<100	<100	<100
	Omicron BA.1	<100	<100	<100	<100	<100	<100	<100	<100	<100	<100	<100	<100	<100	<100	<100	<100	<100	<100	<100	<100
	Omicron BA.4/5	<100	<100	<100	<100	<100	<100	<100	<100	<100	<100	<100	<100	<100	<100	<100	<100	<100	<100	<100	<100
	SARS-CoV	<100	173	<100	<100	<100	<100	<100	<100	<100	108	5131	10594	12259	10946	3208	3145	8095	3445	4533	4717
	Pangolin GD	2330	955	3394	1490	1481	<100	389	291	459	1866	555	1090	938	1851	1202	145	1169	1152	1234	549
	SHC014	<100	<100	<100	<100	<100	<100	<100	<100	108	<100	245	307	234	2071	1163	184	1090	408	587	491
	WIV1	<100	147	<100	<100	<100	<100	<100	<100	<100	<100	2309	5506	4040	3968	3115	1520	6419	486	2423	1363
Week 6	Ancestral	59588	113524	117472	25664	59704	22694	27576	21771	31999	53699	25007	8406	16753	13623	30393	9540	16714	7078	4081	15234
	Alpha	46243	94174	223027	18688	63123	19726	19780	27563	17542	77445	23615	86648	7589	13045	134466	16387	8203	6936	4531	3141
	Beta	9346	43931	13414	2134	3671	2320	3673	8021	17370	13860	1565	9492	6358	5524	10328	5970	7782	3277	3705	10382
	Gamma	42481	60279	150169	32541	43827	15475	12815	21468	24842	31456	29572	50442	33072	32869	42454	85808	13950	14950	19141	13791
	Delta	10962	12568	36366	15669	13706	4465	5251	8573	3225	13330	8220	4918	2743	4442	16044	6993	6561	5619	2363	4665
	Omicron BA.1	1859	5488	14218	344	1394	1194	1047	2885	2456	1394	1588	1818	3732	4863	1744	2684	4400	266	565	1233
	Omicron BA.4/5	885	28661	6521	136	562	192	690	317	482	2672	336	197	841	466	290	2065	559	327	209	1722
	SARS-CoV	3346	32846	13004	9804	7505	6310	13804	19498	39811	29512	478630	257040	75858	46773	204174	234423	281838	707946	181970	169824
	Pangolin GD	246409	265155	336579	95942	726138	36739	50330	106958	393525	203335	62747	55740	132386	147261	123023	62947	130193	39941	65639	103916
	SHC014	2151	2546	1174	335	2146	5337	1099	2303	5754	2645	3239	6432	17734	16590	19857	5550	17606	14418	9403	15798
	WIV1	80276	67141	28143	12858	16355	18310	25281	32778	120950	67137	363776	761702	160520	245228	266808	215654	460640	237994	88354	134912
Week 14	Ancestral	153615	149943	118402	58533	70204	17408	170076	84310	64352	159695	34494	46863	65416	35487	52465	23841	95274	13551	28459	15953
	Alpha	117929	83690	59177	59218	38938	14989	138119	15614	52212	61276	44443	26534	31279	24447	96832	16353	39761	13511	17407	18870
	Beta	67176	342660	12755	60371	22586	6510	41368	22952	25415	65433	39366	46427	31532	38378	26897	12602	64512	8586	12148	19402
	Gamma	121806	44294	33126	79983	74395	23718	72180	55440	36377	91465	23099	57520	65273	28910	55025	19781	46864	42543	18255	14247
	Delta	64745	38843	32799	26735	34039	6227	60643	29267	23505	62614	14225	27519	14022	25725	26517	11880	21503	9765	9874	9333
	Omicron BA.1	39334	28084	23595	3998	12499	5748	11118	7474	14631	17329	5839	25987	90048	18132	18819	13430	5675	3649	2943	4071
	Omicron BA.4/5	15294	111736	17451	9164	10028	449	13600	4082	3306	34961	3192	13189	6357	2114	6822	1846	6806	2245	1704	5439
	SARS-CoV	40738	37154	10715	9120	9120	22387	186209	69183	32359	64565	75858	724436	134896	93325	776247	239883	288403	131826	107152	141254
	Pangolin GD	244818	145315	364113	67623	179194	113768	450134	259763	226544	400101	324534	220530	128841	235903	257194	152510	246840	112076	104633	88408
	SHC014	3397	5639	1434	2584	4303	2845	7085	12254	29764	14649	17837	4473	4649	11152	19746	11635	21096	14994	3372	12628
	WIV1	30519	47591	33612	10378	10598	4414	7619	11593	51327	23720	105695	238901	117934	117064	135574	194387	470162	273039	278213	253442

Legend (colour)	Range
green	<100
yellow	101-1,000
orange	1,001-10,000
red	10,001-100,000
magenta	100,001-1,000,000
